# Consistency Rates of Clinical and Histopathologic Diagnoses of Oral Soft Tissue Exophytic Lesions

**DOI:** 10.5681/joddd.2009.022

**Published:** 2009-09-16

**Authors:** Javad Sarabadani, Maryam Ghanbariha, Saeedeh Khajehahmadi, Masoumeh Nehighalehno

**Affiliations:** ^1^Assistant Professor, Department of Oral Medicine, Faculty of Dentistry and Dental Research Center, Mashhad University of Medical Sciences, Mashhad, Iran; ^2^Assistant Professor, Department of Oral and Maxillofacial Pathology, Faculty of Dentistry, Zahedan University of Medical Sciences, Zahedan, Iran; ^3^Assistant, Department of Oral and Maxillofacial Pathology, Faculty of Dentistry, Mashhad University of Medical Sciences, Mashhad, Iran; ^4^Dentist, Private Practice, Mashhad, Iran

**Keywords:** Clinical diagnosis, exophytic lesion, histopathologic diagnosis

## Abstract

**Background and aims:**

Histpathologic diagnosis of exophytic lesions is occasionally influenced by clinical and radiograph-ic diagnosis and even the surgeon’s observation during biopsy. The aim of this study was to evaluate the cases with failure in clinical diagnosis.

**Materials and methods:**

A total of 73 patients with peripheral exophytic lesions were evaluated in Zahedan Faculty of Den-tistry in 2006. Specialists gave their differential diagnoses based on the criteria of oral medicine texts. Then a biopsy was taken and the histopathologic diagnosis was determined. Finally, consistency rates of clinical and histopathologic diagnoses were de-termined. Statistical analysis was carried out with SPSS software using Chi-Square and Fisher’s exact tests.

**Results:**

In the present study 73 subjects with oral soft tissue (peripheral) exophytic lesions were orally examined and biopsies were taken. Forty-four subjects (60.35%) were females and 29 (39.7%) were males. A total of 81.7% (62 subjects) of clinical diagnoses were consistent with histopathologic reports. In 18.3% (11 subjects) of the cases clinical diagnoses were not con-firmed by histopathologic reports.

**Conclusion:**

In order to reach a diagnostic agreement, conformity of clinical and histopathologic diagnoses is necessary.

## Introduction

The oral cavity and jaws can be the location of many diseases including exophytic lesions with a prevalence of 25.8%,^1^ which may arise from osseous (central) or extraosseous (peripheral) tissues. The term exophytic lesion means any pathologic growth that projects above the normal contours of the oral surface.^2^ Exophytic lesions are often difficult to diagnose clinically due to different histopathologic processes, which can lead to similar lesions. For example, tumors appear similar to cysts, hyperplasia similar to tumors, and benign tumors similar to malignant types.

For correct diagnosis obtaining medical history, dental history and physical examination of the oral cavity (inspection, palpation, percussion and auscultation) are necessary.^3^ Although the histopathologic diagnosis is the basis of treatment for most lesions, comprehensive radiographic and clinical evaluation is required to reach a definite diagnosis.^4 , 5^

However, occasionally, a surgeon does not obtain the specimen from a proper level; therefore, the nature of the lesion cannot be identified. In such cases, biopsy should be taken from the deeper parts of the lesion.^4^ Similarities in clinical, radiographic and microscopic characteristics of some oral exophytic lesions give rise to some difficulties in the proper diagnosis of exophytic lesions. The aim of the present study was to evaluate the cases with failure in clinical diagnoses.

## Materials and Methods

In this descriptive cross-sectional study, 73 patients with peripheral exophytic lesions were evaluated in the Department of Oral Medicine, Faculty of Dentistry, Zahedan University of Medical Sciences, in 2006.

The sample size in this study was estimated according to the ratio estimation in a community. In addition, preliminary studies showed that on average 7 patients suffering from peripheral exophytic lesions were referred to Zahedan Faculty of Dentistry every month. Therefore, 73 patients with peripheral oral exophytic lesions were evaluated in this study, considering P = 0.5, α = 0.05, and d = 0.1. Specialists gave their differential diagnoses based on the criteria of oral medicine references. Moreover, if necessary, laboratory tests, aspirations, and occasionally complementary radiographs were taken from each subject.

After biopsy, the specimens were sent to the Oral Pathology Department at Zahedan Faculty of Dentistry for histopathologic diagnosis. Then the consistency rates for clinical diagnosis were defined by histopathologic diagnosis (gold standard). Statistical analysis was carried out with SPSS software, using Chi-Square and Fisher’s exact tests.

## Results

In the present study 73 subjects with oral soft tissue (peripheral) exophytic lesions were evaluated; 44 subjects were females (60.3%) and 29 were males (39.7%). The subjects were orally examined and biopsies were taken. Female subjects were 8-80 years old (with a mean age of 32) and male subjects were 5-80 years old (with a mean age of 43).The duration of lesions in females was between 14 days and 5 years (mean = 10 months) and in males between 21 days and 10 years (mean = 16 months).

A total of 81.7% (62 subjects) of clinical diagnoses were consistent with histopathologic reports. In 18.3% (11 subjects) the clinical diagnosis was not confirmed histopathologically (Table 1).

**Table 1:** Subjects whose clinical diagnosis was not confirmed by histopathologic report

	Clinical Diagnosis	Histopathologic Diagnosis	Location
1	Verrucous Vulgaris	Irritation Fibroma	Maxillary Facial Gingiva
2	Verrucous Carcinoma	Squamous Cell Carcinoma (SCC)	Floor of the Mouth
3	Peripheral Giant Cell Granuloma (PGCG)	Pyogenic Granuloma (PG)	Mandibular Gingiva
4	Verrucous Carcinoma	SCC	Mandibular Gingiva
5	Verrucous Carcinoma	SCC	Maxillary Facial Gingiva
6	PG	PGCG	Buccal Mandibular Gingiva
7	Verrucous Carcinoma	SCC	Maxillary Gingiva
8	PGCG	PG	Maxillary Gingiva
9	SCC	Verrucous Carcinoma	Mandibular Gingiva
10	SCC	Verrucous Carcinoma	Lower Lip
11	Verrucous Carcinoma	SCC	Mandibular Gingival

The greatest consistency was observed for pyogenic granuloma (22 cases), whereas squamous cell carcinoma (SCC) and verrucous carcinoma (7 cases) revealed the least consistency.

## Discussion

The aim of this study was to identify the cases with failure in clinical and histopathologic diagnoses.

In the present study histopathologic diagnoses confirmed initial clinical diagnoses in 62 (81.7%) but did not do so in 11(18.3%) subjects.

Oral medicine focuses on diagnosis and treatment of oral soft tissue lesions and represents the clinical arm of oral pathology while oral pathology deals with microscopic diagnosis of oral maxillofacial lesions. ^6^

There are not any exactly similar studies. However, Sardellah et al ^7^ compared the accuracy rates of oral medicine prior to referring the patients with histopathologic diagnoses to an Oral Medicine Department. It was a retrospective investigation on the patients’ referral forms from 2005 to 2007, conducted by family physicians with no dental degree, other categories of physicians, and general dental practitioners. Of 678 subjects, 305 (45%) had clinical diagnoses and no radiographic diagnoses of lesions had been given. Finally, it was purported that Italian physicians and dentists had limited information in oral medicine field.^7^

Deihimi et al ^3^ worked on old files in a retrospective study in which only the title was somehow similar to this study. Thirty-four of them did not have definite clinical or histopathologic diagnosis. In fact, only the accuracy rates of clinical diagnoses with histopathologic diagnoses were consistent, although the authors did not mention the types of misdiagnosis and the reasons for that.

Sometimes there are controversies over definite pathologic reports among oral pathologists, which lead to difficulties in treatment planning.

Abbey et al ^8^ evaluated 6 dentists on the Oral Pathology Board in order to determine the histologic diagnoses of 120 oral specimens. Their diagnoses varied from simple hyperkeratosis to severe dysplasia. The agreement, when final diagnosis was mild to moderate dysplasia, was only 50.5% while these pathologists gave only a 50.8% approval in their re-investigations. Approximately in 20% of the subjects, pathologists could not confirm their previous opinions regarding presence of dysplasia.^8^

Powsner et al ^9^ showed surgeons had an improper concept from pathology reports in 30% of the cases. Surgical experience and better cooperation between surgeons and pathologists reduce this gap.

Basically, the ideal to reach a final diagnosis depends on the evaluation of all the clinical and radiographic findings and histopathology of the lesion, leading to a diagnostic agreement, acceptable to all.

Clinical diagnosis of some exophytic lesions necessitates radiographic interpretation. It is followed by removal of bone from the upper layer of the lesion for biopsy by a surgeon and determining its exact location and nature. In some subjects, this occurs superficially and only from epithelial changes located on the surface of submucosal and non-epithelial lesion (pseudoepithelial hyperplasia) in which the probability of SCC report is high.^10^

Improper clinical diagnosis in this investigation was due to similarities between SCC and verrucous carcinoma (7 subjects), pyogenic granuloma and peripheral giant cell granuloma (3 subjects), and finally a peripheral lesion with irregular surface with a histopathologic report of fibroma but clinically misdiagnosed as verrucous vulgaris (Table 1).

It has also been reported in other studies that there are many similarities among exophytic lesions. Such similarities can be seen in comparing with SCC and verrucous carcinoma, pyogenic granuloma and peripheral giant cell granuloma, respectively.

In a well-developed case of verrucous carcinoma, the clinical pathologic diagnosis is relatively easy to understand.^11^ A differential diagnosis would also include papillary squamous cell carcinoma which resembles verrucous carcinoma.^11^ Verrucous carcinoma, which is characterized by a bulbous growth that pushes into the underlying stroma rather than invading it, is typical of SCC.^12^ It is a low-grade variation of SCC.^13 , 14^

Interestingly, in about 20% of the cases, histopathologically identifiable foci of SCC occur within a lesion that look otherwise like a verrucous carcinoma. These hybrid (verrucous-squamous) tumors are said to be associated with a higher recurrence rates than pure verrucous carcinomas.^3^

Peripheral giant cell granuloma is, for all practical purposes, a site-specific variant of pyogenic granuloma (Figure 1).^13^ Generally, this lesion is clinically indistinguishable from a pyogenic granuloma and biopsy findings are definitive in establishing the diagnosis.^11^ Therefore, 98.85 (72 subjects) of clinical diagnoses were consistent with histopathologic reports and in 1.3% (1 subject) the clinical diagnosis was not confirmed histopathologically.


Figure 1. (a) Pyogenic granuloma: clinical diagnosis was peripheral giant cell granuloma. (b) Peripheral giant cell granuloma: clinical diagnosis was pyogenic granuloma.

**a:**
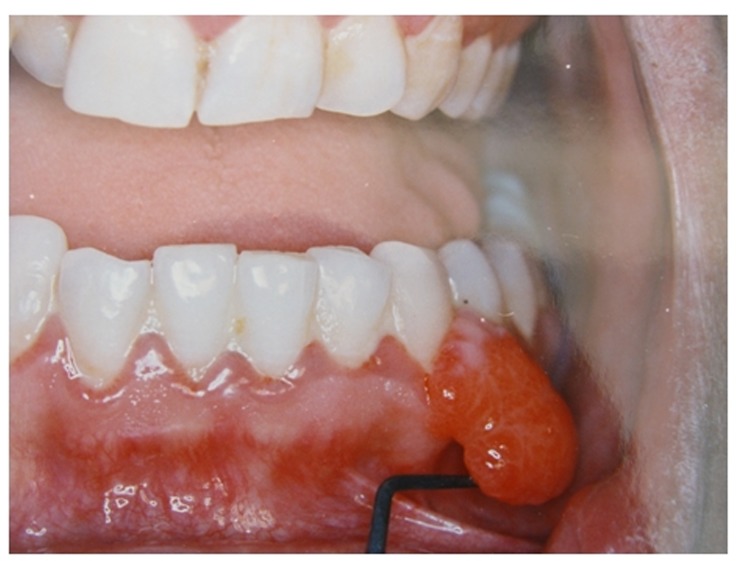

**b:**
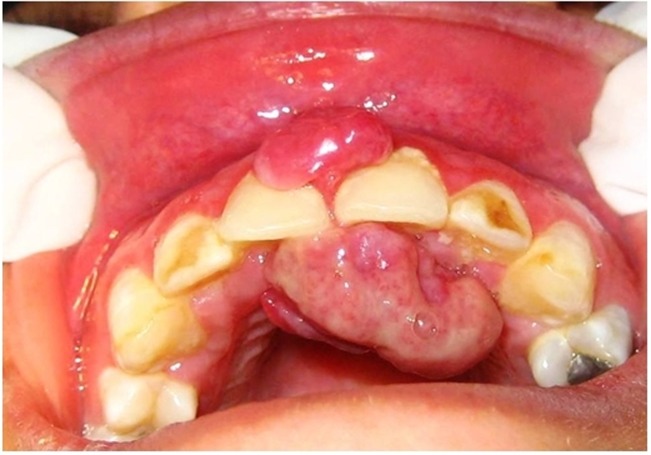

## Conclusion

The clinical, radiographic, and histopathologic similarities between various oral and jaw exophytic lesions sometimes make the diagnostic agreement impossible. Moreover, expert specialists can arrive at the best treatment plan when considering the importance of lesion characteristics. According to some failures reported in clinical diagnosis, attention to details in clinical examination and taking history is recommended to reach a correct diagnosis.
